# Interrelationship between micronutrients and cardiovascular structure and function in type 2 diabetes

**DOI:** 10.1017/jns.2021.82

**Published:** 2021-10-04

**Authors:** Grace W. M. Walters, Emma Redman, Gaurav S. Gulsin, Joseph Henson, Stavroula Argyridou, Thomas Yates, Melanie J. Davies, Kelly Parke, Gerry P. McCann, Emer M. Brady

**Affiliations:** 1 Department of Cardiovascular Sciences, University of Leicester, Leicester, UK; 2 National Institute for Health Research (NIHR) Leicester Biomedical Research Centre, Leicester, UK; 3 Diabetes Research Centre, University of Leicester, Leicester, UK; 4 University Hospitals of Leicester NHS Trust, Leicester, UK

**Keywords:** Cardiovascular function, CMR, Low calorie, Low-energy meal replacement plan, Micronutrients, Type 2 diabetes, BMI, body mass index, CMR, cardiac magnetic resonance imaging, CVD, cardiovascular disease, EF, ejection fraction, HF, heart failure, LV, left ventricular, MRP, meal replacement plan, PLP, pyridoxal 5-phosphate, RCT, randomised control trial, T2D, type 2 diabetes

## Abstract

Micronutrients are important for normal cardiovascular function. They may play a role in the increased risk of cardiovascular disease observed in people with type 2 diabetes (T2D) and T2D-related heart failure. The aims of this study were to (1) examine micronutrient status in people with T2D *v.* healthy controls; (2) assess any changes following a nutritionally complete meal replacement plan (MRP) compared with routine care; (3) determine if any changes were associated with changes in cardiovascular structure/function. This was a secondary analysis of data from a prospective, randomised, open-label, blinded end-point trial of people with T2D, with a nested case–control [NCT02590822]. Anthropometrics, cardiac resonance imaging and fasting blood samples (to quantify vitamins B_1_, B_6_, B_12_, D and C; and iron and ferritin) were collected at baseline and 12 weeks following the MRP or routine care. Comparative data in healthy controls were collected at baseline. A total of eighty-three people with T2D and thirty-six healthy controls were compared at baseline; all had micronutrient status within reference ranges. Vitamin B_1_ was higher (148⋅9 *v*. 131⋅7; *P* 0⋅01) and B_6_ lower (37⋅3 *v*. 52⋅9; *P* 0⋅01) in T2D *v*. controls. All thirty participants randomised to routine care and twenty-four to the MRP completed the study. There was an increase in vitamins B_1_, B_6_, D and C following the MRP, which were not associated with changes in cardiovascular structure/function. In conclusion, changes in micronutrient status following the MRP were not independently associated with improvements in cardiovascular structure/function in people with T2D.

## Introduction

Type-2 diabetes (T2D) is a global epidemic, with the current estimated prevalence in the UK being 4⋅7 million (of which 90 % is T2D)^(1)^. T2D is a progressive and chronic disease of which cardiovascular complications are leading causes of morbidity and mortality. It is characterised by dysglycaemia, resultant from a combination of insulin resistance and impaired insulin secretion, and confers a two- to four fold increased risk of heart failure (HF)^(2)^ even after adjustment for the prevalence of other traditional risk factors, such as sex, age, hypertension, coronary artery disease, and dyslipidaemia, which are inherent among people with T2D^(3)^. The structural and functional cardiovascular abnormalities typically described in T2D include concentric left ventricular (LV) remodelling and diastolic dysfunction, which are evident in asymptomatic individuals and recognised as indicators of early or impending HF^(4)^ (American Heart Association (AHA) stage B^(5,6)^).

Multiple micronutrients are essential for normal cardiovascular function, a number of which are involved in pathways that modulate inflammation and oxidative damage and thus are thought to play a role in reducing cardiovascular risk^(7)^. Dietary micronutrient deficiency is common in adults with HF^(8)^. Micronutrients are necessary cofactors for normal cardiac metabolism, and deficiencies have been implicated in the development and progression of HF^(9)^.

Deficiencies in a variety of micronutrients have also been associated with the development and progression of T2D. Vitamin B_1_ is implicated in insulin synthesis and secretion impairment^(10)^ and vitamin B_6_ in insulin resistance^(11)^, and B_12_ deficiency reportedly occurs as a consequence of prolonged metformin use^(12)^. Vitamin D has important effects on insulin action and may impact on a number of pathways with putative importance in the development of T2D^(13)^. As does iron, which plays a direct and causal role in T2D pathogenesis, mediated by both β-cell failure and insulin resistance^(14)^.

There is a clear link between T2D and HF and while the prevalence of T2D among the general population in UK is approximately 7⋅1 %^(15)^, among patients with HF the prevalence of diabetes is estimated to be between 35 and 45 %^(16)^. Given that dietary micronutrient deficiency is common in adults with both HF^(17)^ and obesity^(18)^, along with the purported role of a number of micronutrients in the pathogenesis and progression of T2D^(19)^, and indeed the development of HF; it could be suggested that the association between T2D and HF may be, in part, a result of nutrient deficiencies common to HF and T2D.

Modifiable risk factors, including nutritional alteration, are ever more important with the increasing prevalence of chronic and progressive conditions such as T2D. The identification and correction of micronutrient deficiencies may offer potential therapeutic roles in the prevention and treatment of T2D-related cardiovascular disease (CVD) including HF.

Therefore, the aim of the present paper was to examine circulating levels of vitamin B_1_, vitamin B_6_, vitamin B_12_, vitamin C, vitamin D, iron and ferritin in a cohort of people with T2D with the evidence of subclinical cardiovascular dysfunction. While other micronutrients such as zinc^(20)^ and vitamin E^(21)^ have been associated with heart disease and CVD prevention, these specific micronutrients are not considered in the present study as they were not analysed from the fasting blood samples.

The analysis was a three-part analysis investigating the following: (1) micronutrient status in people with T2D *v*. healthy controls; (2) any changes in micronutrient status following a nutritionally complete meal replacement plan (MRP) compared with routine care and (3) if any changes were associated with changes in cardiovascular structure and function.

## Methods

The rationale, design and main study findings of the ‘Diabetes Interventional Assessment of Slimming or Training to Lessen Inconspicuous Cardiovascular dysfunction’ (DIASTOLIC) trial have been previously reported^(22,23)^. In brief, DIASTOLIC was a prospective, randomised, open-label, blinded end-point trial assessing the effects of a low-calorie MRP *v.* exercise training *v*. routine care in working-age adults with T2D and obesity. This study was conducted according to the guidelines laid down in the Declaration of Helsinki, and all procedures involving human subjects/patients were approved by the National Research Ethics Service (15/WM/0222). All participants provided written informed consent.

### Participants

**Fig. 1.**
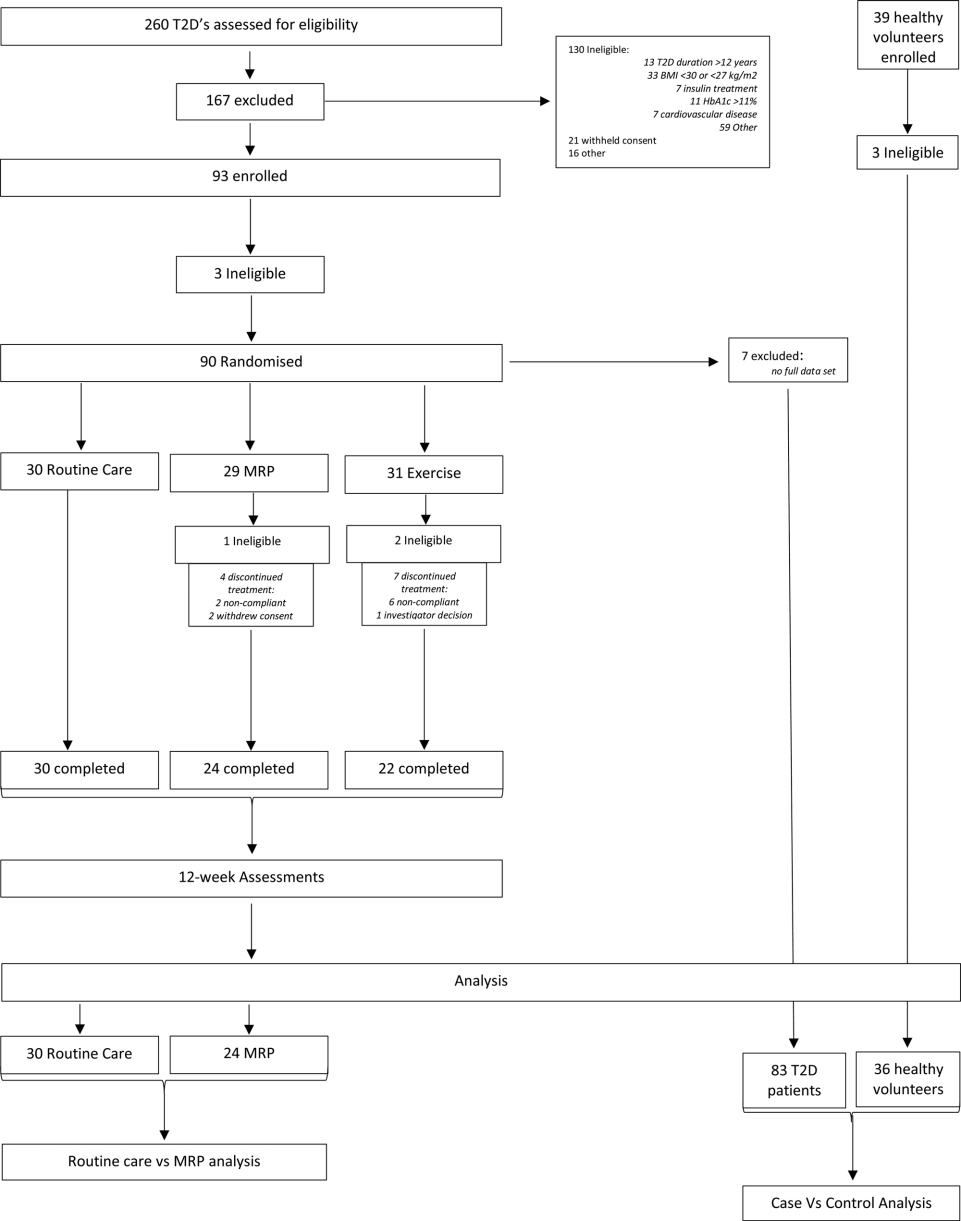
CONSORT diagram. MRP, meal replacement plan; T2D, type 2 diabetes.

The participants included in the DIASTOLIC trial were aged 18–65 years with established T2D (duration >3 months) diagnosed before age 60 years with a body mass index (BMI) of >30 kg/m^2^ (or >27 kg/m^2^ if South Asian or black ethnicity). Participants were ineligible to participate if T2D duration was >12 years, they were on the current treatment of more than three glucose-lowering medications or insulin, had history, signs or symptoms of CVD (including coronary artery disease, stroke, transient ischaemic attack, peripheral artery disease or HF) and had weight loss of >5 kg in the preceding 6 months or an inability to exercise or undertake the MRP. Healthy volunteers free of T2D, obesity, hypertension or confirmed CVD were recruited for baseline case–control comparison. A full list of the inclusion and exclusion criteria is provided within Supplementary Material, Document 1. Those participants who fit the inclusion criteria were randomised to one of the three arms of the intervention group. Age-, sex- and ethnicity-matched healthy volunteers were also recruited for a one time point data collection (equivalent to baseline) for baseline case–control comparisons. See the CONSORT diagram (Fig. 1) for participant numbers enrolled, completion of randomised control trial (RCT) and numbers included in each analysis.

### Interventions

The DIASTOLIC study had three trial arms: routine care, a nutritionally complete MRP and a supervised aerobic exercise programme; details of which can be found in Supplementary Material, Document 2. Participants were randomised (1:1:1) to one of these groups for 12 weeks. For this secondary analysis, we only focused on the routine care and MRP arms because the exercise arm did not alter participants’ micronutrient intake as part of that intervention. The dietary arm followed a low-energy, nutritionally complete MRP, providing all the reference nutrient intakes of micronutrients^(24)^, for 12 weeks which comprised an average of ~810 kcal/d (30 % proteins, 50 % carbohydrates and 20 % fats) (Cambridge Weight Plan).

### Assessments

Participants with T2D in the trial underwent two main assessment visits: baseline and 12 weeks. Each visit consisted of the following assessments: demographics, medical history, bio-anthropometrics (height, weight, BMI, hip:waist ratio, fasting blood sample and blood pressure), comprehensive cardiovascular magnetic resonance (CMR) imaging and a symptom-limited incremental cardio-pulmonary exercise test. The data collected were identical for healthy volunteers and participants with T2D and obesity.

### Fasting blood sample analysis and micronutrient quantification

A fasting venous blood sample was obtained to assess the biochemical profiles for T2D control (glucose and HbA1c) and micronutrients. All fasting blood samples were analysed in an NHS accredited laboratory, and the details of this biochemical analysis can be found in Supplementary Material, Document 3.

### Cardiovascular magnetic resonance

In addition, participants had comprehensive CMR imaging on a 1⋅5 T field strength scanner (MAGNETOM Aera; Siemens, Erlangen, Germany) using a standardised protocol^(22)^. CMR images were analysed offline while blinded to all participant details and the treatment group by experienced operators.

### Statistical analysis

Statistical analyses were performed using a commercially available software package version 26 (IBM SPSS Statistics, Hampshire, UK). The normality of the outcome measures was assessed using histograms, the Shapiro–Wilk test and Q–Q plots. Independent samples *t* tests or Mann–Whitney tests were employed to determine any differences in micronutrients between cases and controls. Data were split according to the treatment group and paired samples *t* tests or Wilcoxon test were used to determine if micronutrients were different from baseline to 12 weeks within the MRP and the routine care group. Independent samples *t* tests or Mann–Whitney tests were then used to determine if the change from baseline to 12 weeks was different between the MRP group and the routine care group. To determine if micronutrient status was correlated with cardiac structure and function variables, bivariate Spearman's correlation estimates were produced using baseline data for the cohort of participants with obesity and T2D. The cardiovascular structure/function variables of interest were those that had previously been described as being improved following the 12-week MRP^(23)^:

1. Change in LV mass to volume ratio (LV mass:volume, g/ml).
2. Change in mean aortic distensibility (mmHg^−1^ × 10^−3^).

The micronutrients that were significantly different from baseline to 12 weeks in the MRP arm only were modelled (using linear regression) against the cardiac structure/function variables. An unadjusted, and baseline-adjusted, univariate model was used to assess if there was any association between changes in micronutrients and changes in cardiovascular structure/function variables. Data are expressed as mean (± standard deviation) for normally distributed data and median (interquartile range) for not normally distributed data. A *P* value <0⋅05 was considered statistically significant.

## Results

A total of eighty-three people with T2D and obesity and thirty-six healthy volunteers were included in the case *v*. control baseline analysis (Table 1). See the CONSORT diagram (Fig. 1) for participant numbers enrolled, completion of RCT and numbers included in each analysis. Intervention groups were well balanced with no significant differences between people in the MRP or routine care arms at baseline, for age, sex, ethnicity, body weight, BMI or HbA1c concentration, or any of the micronutrients at baseline (Table 2). Of the eighty-three participants in the trial, a total of seventy-six completed the intervention, fifty-four of which were in either the MRP (*n* 24) or routine care arm (*n* 30) with no significant differences in characteristics at baseline (Table 2).

**Table 1.** Baseline demographics: cases *v*. controls

Variable	Cases		Controls				
Demographics	Mean or *median*	sd or (IQR)	Mean or *median*	sd or (IQR)			
					*P* value		
*N*	83	N/A	36	N/A	N/A		
Age	50⋅4	6⋅4	48⋅6	6⋅2	0⋅150		
Sex (% male)	57⋅8 %	N/A	52⋅8 %	N/A	0⋅552		
Minority ethnicity (*N* (%))	33 (39 %)	N/A	12 (33 %)	N/A	0⋅51		
T2D duration (months)	62⋅4	38⋅2	N/A	N/A	N/A		
HbA_1c_ (mmol/mol)	7⋅3	1⋅0	5⋅4	0⋅2	<0⋅001		
HbA_1c_ (%)	56⋅0	11⋅2	35⋅3	3⋅6	<0⋅001		
Weight (kg)	102⋅4	16⋅0	70⋅4	10⋅8	<0⋅001		
BMI (kg/m^2^)	36⋅4	5⋅4	24⋅5	2⋅4	<0⋅001		
SBP (mmHg)	139⋅9	15⋅5	120⋅9	13⋅2	<0⋅001		
DBP (mmHg)	87⋅6	8⋅0	76⋅4	7⋅2	<0⋅001		
Heart rate (BPM)	74⋅2	9⋅8	61⋅7	9⋅8	<0⋅001		
							
Cardiovascular variables							
LV mass:volume ratio (g/ml)	0⋅8	0⋅1	0⋅71	0⋅1	<0⋅001		
Aortic distensibility (mmHg^-1^ × 10^−3^)	4⋅2	2⋅1	6⋅6	2⋅0	<0⋅001		
							
Vitamins					Mean difference	(95 % CI)	*P* value
Vitamin B_1_ (nmol/l)	148⋅9	30⋅6	131⋅0	(25⋅0)	17⋅2	(5⋅4, 28⋅9)	0⋅01
Vitamin B_6_ (nmol/l)	32⋅0	(21⋅3)	52⋅9	23⋅8	15⋅7	(24⋅9, 6⋅4)	0⋅01
Iron (μmol/l)	14⋅5	(7⋅0)	16⋅9	6⋅9	1⋅9	(4⋅2, 0⋅4)	0⋅10
Vitamin D (nmol/l)	3⋅0	(2⋅0)	3⋅0	(1⋅0)	0⋅2	(0⋅6, 0⋅1)	0⋅23
Vitamin C (μmol/l)	56⋅7	(39⋅3)	47⋅7	20⋅0	6⋅3	(2⋅9, 15⋅6)	0⋅17
Vitamin B_12_ (ng/l)	390⋅1	136⋅4	374⋅5	(151⋅3)	10⋅9	(67⋅5, 45⋅7)	0⋅83
Ferritin M (μg/l)	119⋅5	(92⋅8)	121⋅0	(88⋅0)	18⋅4	(50⋅4, 87⋅2)	0⋅82
Ferritin F (μg/l)	46⋅0	(67⋅3)	47⋅2	31⋅8	11⋅9	(13⋅2, 37⋅0)	0⋅41

Abbreviations: BPM, beats per minute; DBP, diastolic blood pressure; HbA_1c_, glycated haemoglobin; mmHg, millimetre of mercury; SBP, systolic blood pressure; T2D, type 2 diabetes; sd, standard deviation; CI, confidence interval.

**Table 2.** Baseline demographics: intervention groups

Variable	Routine care		MRP		*P* value
Demographics	Mean	sd	Mean	sd	
*N*	30		24		
Age	50⋅7	6⋅4	51⋅1	5⋅7	0⋅81
Sex (male)	60 %		63 %		0⋅86
Ethnicity (*N* (%)) (White/BAME)	18 (60 %)		15 (63 %)		0⋅86
T2D duration (months)	60⋅5	31⋅5	58⋅3	39⋅8	0⋅39
HbA1c (mmol/mol)	7⋅3	0⋅9	7⋅2	1⋅1	0⋅39
Weight (kg)	102⋅6	14⋅9	106⋅7	16⋅2	0⋅35
BMI (kg/m^2^)	36⋅8	4⋅8	37⋅3	5⋅8	0⋅75
Resting SBP (mmHg)	137⋅8	12⋅7	145⋅9	15⋅9	0⋅05
Resting DBP (mmHg)	85⋅3	7⋅3	91⋅1	7⋅4	0⋅01
Resting heart rate (BPM)	76⋅3	7⋅5	73⋅1	8⋅6	0⋅15
					
Cardiac structure/function					
LV mass:volume ratio (g/ml)	0⋅8	0⋅1	0⋅8	0⋅1	0⋅54
Aortic distensibility (mmHg –1 × 10^−3^)	4⋅2	1⋅9	3⋅7	1⋅9	0⋅22

Abbreviations: BAME, black and minority ethnic; BMI, body mass index; BPM, beats per minute; DBP, diastolic blood pressure; SBP, systolic blood pressure; T2D, type 2 diabetes; sd, standard deviation; IQR, interquartile range.

Only participants with a full data set at baseline were included in the analysis; therefore, three participants with T2D and obesity were excluded from the case *v*. control baseline analysis due to missing data.

### Case–control comparison

The healthy volunteer (control) and T2D with obesity group (case) were similar for age, sex and ethnicity distribution; however, the control group had lower overall body weight and BMI, blood pressure, heart rate and HbA1c (Table 1). Micronutrient levels for both groups were within reference ranges (Supplementary Table S1)^(25,26)^.

Vitamin B_1_ was higher in cases compared with controls (148⋅87 ± 30⋅64 *v*. 131⋅71 ± 25⋅25 nmol/l, *P* 0⋅01), while levels of vitamin B_6_ were lower in cases compared with controls (37⋅28 ± 22⋅01 *v*. 52⋅94 ± 23⋅83 nmol/l, respectively; *P* 0⋅001) (Table 1). Vitamin C, vitamin B_12_, vitamin D, iron and ferritin showed no significant differences between cases and controls (Table 1).

### Pre- and post 12-week MRP

Twelve-week changes in bio-anthropometrics, LV mass:volume and aortic distensibility in the two trial arms are presented in Supplementary Table S1 and Document 4. In brief, following the 12-week MRP, body weight was reduced, and blood pressure, glycaemic status, LV mass:volume ratio and aortic distensibility all improved (all *P* < 0⋅05). The routine care arm also showed a significant reduction in weight (median [IQR] −1⋅05 (−3⋅16, −0⋅01)); however, this was a significantly smaller reduction than the weight reduction seen in the MRP arm. Blood pressure was also reduced in the routine care arm (mean −7⋅1 mmHg systolic, −1⋅8 mmHg diastolic). No significant changes in cardiovascular structure and function measures were observed following 12 weeks of routine care.

**Fig. 2.**
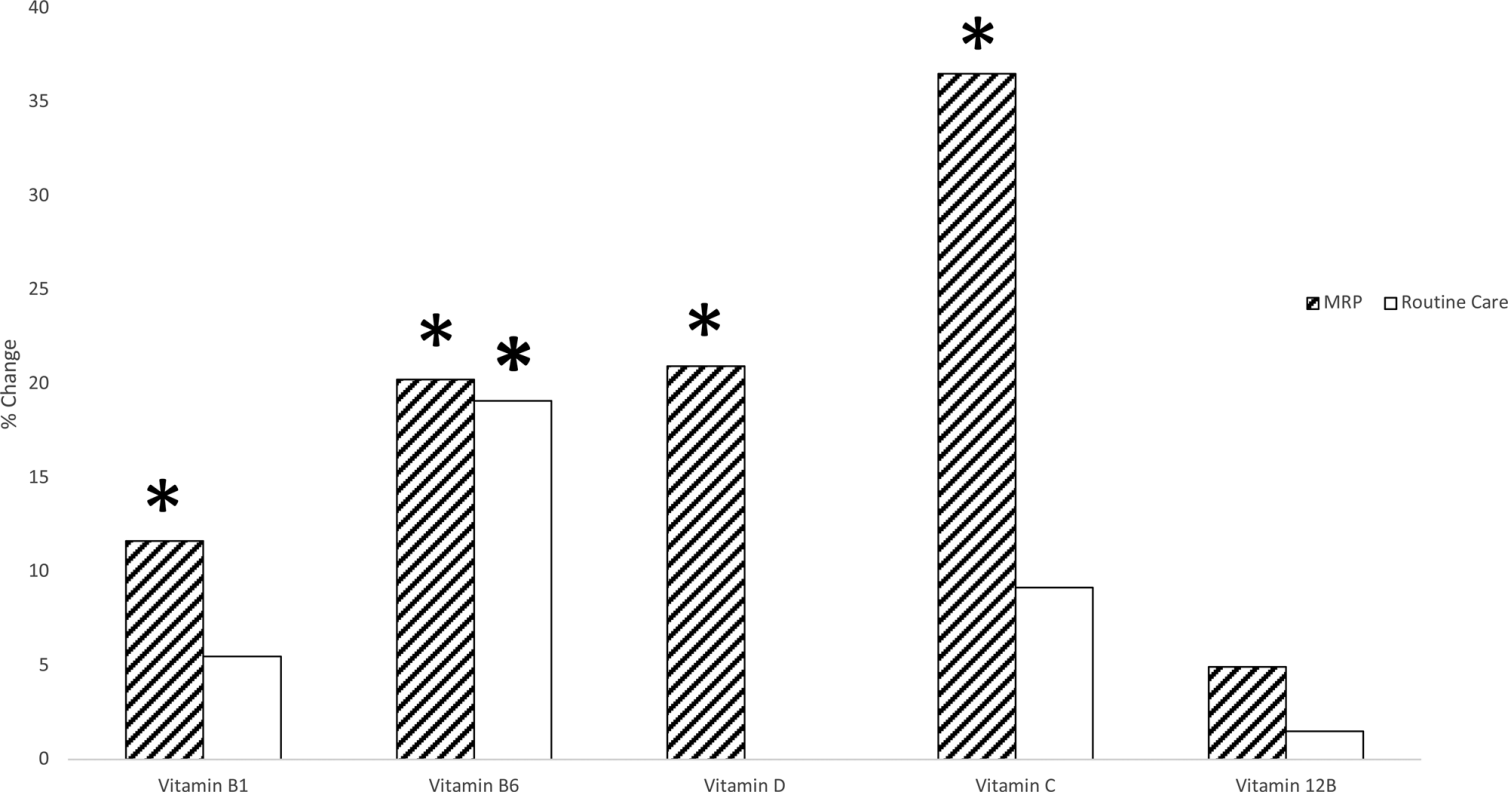
Comparison of between groups (MRP and routine care) change in micronutrients from baseline to 12 weeks. Data split according to the treatment group. Paired samples *t* tests or Wilcoxon test were used to determine if micronutrients were different from baseline to 12 weeks within the MRP and the routine care group. Independent samples *t* tests or Mann–Whitney tests were then used to determine if the change from baseline to 12 weeks was different between the MRP group and the routine care group. *Represents a significant change from baseline to 12 weeks. MRP, meal replacement plan.

Four micronutrients increased from baseline to 12 weeks following the MRP (Table 3): vitamin B_1_ (+16⋅38 [± 24⋅20]; *P* 0⋅009), vitamin B_6_ (+11⋅5 [27⋅25]; *P* 0⋅016), vitamin D (+0⋅50, [1⋅0]; *P* 0⋅005) and vitamin C (+12⋅59 [41⋅78]; *P* 0⋅009). Vitamin B_12_, ferritin and iron showed no significant differences from baseline to 12 weeks post-MRP. Vitamin B_6_ also increased in the routine care arm (+6⋅00 [±12⋅00]; *P* 0⋅014) (the percentage change within groups is illustrated in Fig. 2).

**Table 3.** Vitamin profile change: routine care and MRP

	Baseline		12 weeks		Change		
Variable	Mean or *median*	sd or (IQR)	Mean or *median*	sd or (IQR)	Mean or *median*	sd or (IQR)	*P* value
Routine care							
Vitamin B_1_ (nmol/l)	152⋅48	31⋅01	160⋅87	27⋅15	8⋅39	22⋅51	0⋅13
Vitamin B_6_ (nmol/l)	*28*⋅*00*	(23⋅00)	*38*⋅*00*	(18⋅25)	*6*⋅*00*	(12⋅00)	0⋅01
Iron (μmol/l)	*14*⋅*00*	(7⋅00)	*12*⋅*50*	(7⋅75)	−1⋅00	(5⋅00)	0⋅31
Vitamin D (nmol/l)	*3*⋅*00*	(2⋅00)	*3*⋅*00*	(1⋅00)	*0*⋅*00*	(2⋅00)	0⋅81
Vitamin C (μmol/l)	38⋅87	23⋅96	41⋅43	22⋅05	4⋅01	16⋅08	0⋅190
Vitamin B_12_ (ng/l)	*345*⋅*00*	(111⋅50)	*330*⋅*00*	(164⋅50)	*0*⋅*00*	(93⋅00)	0⋅461
Ferritin M (μg/l)	*113*⋅*50*	(106⋅00)	*99*⋅*00*	(85⋅75)	*−13*⋅*50*	(57⋅50)	0⋅56
Ferritin F (μg/l)	*45*⋅*00*	(45⋅00)	*51*⋅*00*	(64⋅50)	2⋅00	(15⋅00)	0⋅72
							
MRP							
Iron (μmol/l)	14⋅31	6⋅02	15⋅31	5⋅48	1⋅00	3⋅39	0⋅332
Vitamin D (nmol/l)	*3*⋅*00*	(2⋅00)	*3*⋅*00*	(1⋅00)	*0*⋅*50*	(1⋅0)	0⋅005
Vitamin C (μmol/l)	*35*⋅*96*	(42⋅71)	*55*⋅*12*	(31⋅59)	*12*⋅*59*	(41⋅78)	0⋅009
Vitamin B_12_ (ng/l)	*402*⋅*00*	(200⋅00)	*430*⋅*00*	(205⋅00)	*43*⋅*00*	(141⋅75)	0⋅10
Ferritin M (μg/l)	*97*⋅*00*	(162⋅00)	*106*⋅*00*	(172⋅00)	*11*⋅*00*	(87⋅00)	0⋅31
Ferritin F (μg/l)	58⋅44	46⋅18	65⋅00	49⋅54	6⋅56	27⋅35	0⋅49

Abbreviations: BAME, black and minority ethnic; BMI, body mass index; BPM, beats per minute; DBP, diastolic blood pressure; SBP, systolic blood pressure; T2D, type 2 diabetes; sd, standard deviation, IQR, interquartile range. Data split according to the treatment group. Paired samples *t* tests or Wilcoxon test were used to determine if micronutrients were different from baseline to 12 weeks within the MRP and the routine care group.

Vitamin D showed a trend towards increasing levels in the MRP arm (*P* 0⋅06). However, all other micronutrients showed no differences in the change from baseline to 12 weeks between the two arms.

There were no significant correlations or linear associations between the change in micronutrients and previously observed changes in cardiovascular structure/function (Supplementary Tables S3 and S4). Due to a lack of a significant relationship between the measures of cardiovascular structure/function with the unadjusted model, the only additional model was that which included baseline adjustment for vitamin level which also demonstrated no significant relationship (data not reported).

## Discussion

To our knowledge, this is the first study to investigate the effects of a nutritionally complete MRP on the plasma micronutrient concentrations of people with obesity and established T2D (but asymptomatic AHA stage B HF^(6)^) and its subsequent impact on cardiovascular structure and function. The major findings of this secondary analysis were that vitamin B_1_ and B_6_ levels are different between people with obesity and T2D compared with matched healthy controls. Secondly, following a 12-week intervention with a low-energy MRP and associated dramatic reductions in weight, blood pressure and glycaemia, there were significant increases in vitamins B_1_, B_6_, D and C. Despite this, these changes were not associated with the previously observed improvements in cardiovascular structure and function.

A key finding from our investigation was that vitamin B_1_ was higher in people with T2D compared with healthy controls. This was unexpected as previously vitamin B_1_ deficiency has been reported among people with T2D and is implicated in the development of various complications of advancing diabetes^(27)^. A cross-sectional comparative study of normal controls showed that thiamine (vitamin B_1_) was lower in people with T2D^(28)^, and several studies have demonstrated thiamine deficiency in individuals with both type 1 and type 2 diabetes^(10)^. Insulin deficiency is associated with a reduction in the rate of thiamine transport across the intestine and thiamine deficiency leads to a marked impairment in insulin synthesis and secretion; thus, insulin deficiency may exacerbate thiamine deficiency and vice versa^(10)^. B vitamins have been postulated to play a role in CVD prevention and cardiovascular health. Thiamine deficiency is a recognised contributor of new-onset HF of initially uncertain aetiology^(29)^ and a meta-analysis of randomised, double-blind, placebo-controlled trials reported that thiamine supplementation in patients with systolic HF increased ejection fraction compared with placebo^(30)^. This discrepancy in levels of vitamin B_1_ between our results and the current literature may be a result of the relatively small sample sizes and the relatively young age and short T2D duration within the present cohort. The participants in this study had an average T2D duration of just 62⋅4 months; this relatively short T2D duration is the most likely result of the participants being younger than in previous studies (the DIASTOLIC study only recruited working-age adults). The higher vitamin B_1_ level among the participants with T2D and obesity is also likely to have limited clinical relevance because vitamin levels for both groups were within the normal range^(25,26)^. It is, however, worth noting that while vitamin B_1_ was within reference ranges, the T2D participants in the MRP arm of the intervention did see a significant increase in vitamin B_1_. However, the change was not significantly different from the change seen in the routine care arm, therefore adding to the narrative that this increase is likely clinically insignificant.

We also observed lower levels of vitamin B_6_ in people with T2D and obesity, which is in line with the previous literature. The deficiency of vitamin B_6_ has been implicated in T2D^(31)^ and reduced vitamin B_6_ availability is implicated in insulin resistance^(32)^. It has been observed that people newly diagnosed with T2D have lower concentrations of the catalytically active part of vitamin B_6_, pyridoxal 5′-phosphate (PLP) compared with people without T2D^(33)^. PLP is involved in the pathway that converts tryptophan into nicotinic acid^(11)^ and metabolites produced when this pathway does not work properly, partly as a consequence of low levels of PLP, can interfere with insulin activity^(11)^, causing insulin resistance, a hallmark of T2D. PLP deficiency may also impact on insulin resistance by controlling the expression of genes involved in adipogenesis^(11)^, or may cause insulin resistance through an increase of homocysteine due to the impairment of enzymes^(11)^. There is also evidence that vitamin B_6_ deficiency may negatively affect the progression of T2D once the disease is present^(27)^, via its contribution to insulin resistance. Insulin resistance and prolonged elevated circulating glucose levels may contribute to the pathogenesis of diabetic cardiomyopathy^(4)^. Insulin resistance and worsening glycaemia are associated with increased LV mass to LV end-diastolic volume ratio (a marker of remodelling) indicating a potential indirect role of B_6_ deficiency in concentric LV remodelling^(34)^. The findings of the present study are, however, in conflict with this as an association between the increase in vitamin B_6_ and the improvement in LV mass:volume ratio was not identified. This lack of association may be a result of the fact that vitamin B_6_ was within healthy reference ranges at baseline and 12 weeks; therefore, while there was a significant increase in vitamin B_6_ following the 12-week MRP, the concentration of vitamin B_6_ was always within a ‘healthy’ range. An association between improvements in vitamin B_6_ and LV mass:volume ratio may be present if the concentration of vitamin B_6_ increased from an ‘unhealthy’ to a ‘healthy’ range; thus, this lack of association may be associated with the fact that participants did not present with sub-optimal nutritional status.

A significant increase in four micronutrients (vitamins B_1_, B_6_, D and C) in the MRP arm was observed. This is the first time to our knowledge that these changes have been observed following a nutritionally complete MRP in people with T2D. This increase was not unexpected as this was a nutritionally complete MRP, providing all the reference nutrient intakes of micronutrients^(24)^. As the observed increase in micronutrients was maintained within reference ranges, it was unlikely to have any clinically significant impact.

The improvement in vitamin D may be of particular importance when considering the role that vitamin D deficiency is postulated to play in the development and/or progression of chronic HF^(35,36)^. Vitamin D deficiency has been shown to be associated with increased LV dilation, assessed by transthoracic echocardiography, in an elderly chronic HF population^(37)^. An LV mass:volume ratio has been shown to be higher (suggestive of concentric LV remodelling) in those with T2D^(38)^. However, there was no difference in levels of vitamin D between cases and controls in the present study, despite vitamin D deficiency being common among this population group^(39)^. This may again be attributed to the health status of the present cohort and may be different for people with a longer duration of, and more poorly controlled, T2D, or those who have additional comorbidities.

Our final finding is that changes in micronutrients were not independently associated with the observed improvements in cardiovascular structure and function, therefore suggesting that there is likely another mechanism(s) responsible for these favourable alterations. Contrary to this, a lack of association could be resultant of the relatively good health status of this particular cohort of participants who were asymptomatic of CVD had a relatively short duration of T2D (62⋅4 months) which was relatively well controlled (mean HbA1c 7⋅27 %), and their circulating micronutrients were within reference ranges. Therefore, there may be a limit to the expected level of improvement. Furthermore, the original trial was not powered to detect significant differences in micronutrients between the groups following the interventions; therefore, the observed results could be resultant from type II error^(40)^. A further limitation of this investigation was that while the participants’ micronutrient supplementation at baseline was captured in the case report forms as part of the medical history, it was not collected robustly. The information provided did not allow us to standardise or confirm what combination of nutrients, quantity, frequency or quality of supplement. Therefore, this was not included as a confounder in the univariate model that was used to assess if there was any association between changes in micronutrients and changes in cardiovascular structure/function variables. This, therefore, warrants further investigation in a larger, sufficiently powered cohort that includes older symptomatic adults, with longer duration or less well-controlled T2D. There is also potential value in identifying people with T2D and obesity with poor nutritional status and investigating the effects of such an intervention.

An alternative mechanism responsible for the improvements in LV mass:volume ratio and aortic distensibility that we observed in the MRP group may be centred on the reversal of the haemodynamic alterations that occur with loss of excess body weight. Weight loss is associated with reductions in many of the haemodynamic alterations that develop with obesity^(41)^. Obesity exerts numerous effects on the cardiovascular system, including changes in cardiac loading, energy substrate utilisation (via insulin resistance), tissue metabolism and systemic inflammation, all of which are believed to individually promote HF progression^(42,43)^. A recent large meta-analysis reports that the likelihood of displaying LV hypertrophy was higher in those with obesity^(44)^. Weight reduction is consistently associated with the decrease in LV mass^(44,45)^. Recent studies propose that improvements in neurohormonal and metabolic abnormalities associated with weight reduction such as sympathetic nervous system tone, renin–angiotensin–aldosterone system activation, insulin resistance and hyperleptinaemia may also play a role in reducing LV mass^(46)^. Indices of diastolic function have also consistently been reported to improve following weight loss^(47)^. Therefore, it could be the significant weight loss observed in the MRP group (−13⋅5 kg) that is driving the favourable cardiovascular changes.

The novelty of this secondary analysis from data from an RCT is that it is the first analysis to investigate the effects of a nutritionally balanced MRP on the plasma micronutrient concentrations of people with T2D and obesity and its subsequent impact on cardiovascular structure and function as determined by CMR. However, this secondary analysis uses a small sample size, and the null findings may be a result of a lack of statistical power (type II error) making it difficult to draw meaningful conclusions. We recommend further work to confirm the findings of the present analysis, which should include cohorts of people who have sub-optimal nutritional status.

## Conclusion

The concentrations of vitamins B_1_ and B_6_ differ between people with T2D and age- and sex-matched healthy controls. Among people with T2D, vitamin D concentration and LV mass:volume ratio are associated at baseline. A 12-week MRP induced a significant increase in several micronutrients (vitamins B_1_, B_6_, D and C). However, the improvement in circulating micronutrient levels showed no association with the observed improvements in cardiovascular structure or function following the 12-week MRP in people with T2D and obesity. This lack of association may be the consequence of the relatively good health status of this cohort and the small sample size. This area of research, therefore, warrants further investigation.

## Supplementary material

For supplementary material accompanying this paper visit http://dx.doi.org/10.1017/jns.2021.82. click here to view supplementary material
