# Supplementary material for: Interrelationship between micronutrients and cardiovascular structure and function in type 2 diabetes
Source: J Nutr Sci. 2021 Oct 4;10:e88. doi: 10.1017/jns.2021.82 (PMC8532075; doi:10.1017/jns.2021.82)
Supplement: Supplementary file 1 [file S2048679021000823sup001.docx]

## Supplementary Document 1: DIASTOLIC inclusion and exclusion criteria

**Inclusion criteria**

- Capacity to provide informed consent before any trial- related activities.
- Established T2D (≥3months).
- HbA1c ≤9% if on triple therapy or ≤10% on diet and exercise or monotherapy or dual therapy.
- Current glucose-lowering therapy either mono, dual, or triple of any combination of metformin, sulphonylurea, DPP-IV inhibitor, GLP-1 therapy or an SGLT2i and diet and exercise.
- Poorly managed diet controlled diabetes (with HbA1c >6.5%, not currently taking any glucose-lowering therapy, meeting BMI inclusion range).
- BMI >30kg/m^2^ or >27kg/m^2^ (if South Asian or black ethnicity).
- Diagnosis of T2D before 60 years of age.
- Age ≥18 and ≤65years.

**Exclusion criteria**

- Diabetes duration >12years.
- Currently taking more than three glucose-lowering therapies.
- Weight loss of >5kg in the preceding 6 months.
- Stage 4 or 5 chronic kidney disease (eGFR <30 mL/min/1.73 m^2^).
- Current therapy with insulin, thiazolidinediones, steroids or atypical antipsychotic medication.
- Untreated thyroid disease.
- Known macrovascular disease including coronary artery disease, stroke/TIA or peripheral vascular disease.
- Presence of arrhythmia (including atrial fibrillation, atrial flutter or second or third degree atrioventricular block).
- Known heart failure or other clinically relevant heart disease.
- Inability to exercise or undertake a MRP.
- Absolute contraindication to CMR.
- Cardiovascular symptoms (angina and limiting dyspnoea during normal physical activity).
- Inflammatory condition, for example, connective tissue disorder and rheumatoid arthritis.

(BMI: body mass index; CMRI: cardiovascular MRI; DPP-IV: dipeptidylpeptidase-IV inhibitor; eGFR: estimated glomerular filtration rate; GLP-1: glucagon-like peptide 1; MRP: meal replacement plan; SGLT2i: sodium glucose cotransporter 2 inhibitor; T2D: type 2 diabetes; TIA: transient ischaemic attack).

Supplementary Document 2: DIASTOLIC details of interventions.

Recruited participants will be randomised to one of three groups (all of which also received health coaching alongside their randomly allocated intervention):

1) Standard of care as per National Institute for Health and Care Excellence guidance^(1)^, including lifestyle advice

(2) MRP

(3) Supervised exercise training

The standard care group were provided with standard lifestyle advice according to National Institute for Health and Care Excellence (NICE) guidance, delivered in a single health coaching interview at week 0 and week 12. The NICE guidance included: patient education, dietary & lifestyle advice, and advice on blood pressure and glucose targets

The MRP group received a low-energy MRP diet containing an average of ≈810 kcal/day (30% protein, 50% carbohydrate, 20% fat) (Cambridge Weight Plan). The diet plan complied with all current guidance and government legislation (European Food Safety Authority) for macronutrient and micronutrient content and quality ^(2)^.

The MRP involved participants being given a tailored meal replacement plan to follow for up to 12 weeks. The meal replacement plan was provided by Cambridge weight plan (Corby, UK).

The MRP group received a total meal replacement diet containing ~810 kcal/day (40% protein, 50% carbohydrate, 10% fat). This diet plan complied with all current guidance and government legislation (food standards agency) for macro- and micro nutrient content and quality^(3)^. Participants were given a plan containing 4 mini-meals per day with an allowance of milk or a non-dairy alternative. The mini meals were either a formula food packet (available in several flavours and textures). In addition, there was a number of different snack bars available. The snack bar was eaten in place of a food pack because it provided the equivalent nutritional composition as a food pack. Participants selected the items that they found most palatable and decided upon what times of the day they would prefer to eat and space the items out. Supplement packs were supplied in small batches over the course of the study, thus allowing each participant to identify those that the find most palatable and enjoyable. Also, the meal replacement plan was supplemented with two portions of non-starchy vegetables and participants were encouraged to drink ~2 litres of water, or other sugar free, zero calorie drinks, per day. Therefore, participants were required to abstain from alcohol for the duration of the study. Participants were provided with a list of accepted non-starchy vegetables and were given information about what constitutes a portion to support their decision making.

The MRP was undertaken alongside health behaviour coaching and relapse prevention through weekly contact, where possible, with a qualified dietician or equivalent.

Participants randomised to the MRP group discontinued all glucose-lowering therapies following randomisation to avoid hypoglycaemia. Antihypertensive drugs were stopped on the day of low-energy diet commencement, as a safety measure because blood pressure was likely to fall substantially (mean drop in systolic blood pressure ~20 mm Hg) on the diet. Any alterations to medication were ultimately made at the discretion of the study clinician(s). The clinical team included specialists in CVD and diabetes medicine to oversee alterations in antihypertensive and glucose-lowering therapy.

## Supplementary Document 3: Biochemical analysis of vitamins and micronutrients

| Test | Assay | Manufacturer | Equipment |
| --- | --- | --- | --- |
| Vitamin D (Total (25 OH) vitamin D) | Competitive Immunoassay | Siemens | ADVIA Centaur |
| Vitamin B1 | External lab (high-performance liquid chromatography [HPLC] with fluorimetric detection) | HPLC system consisted of a Waters solvent delivery system and a Waters fluorimeter, Model 474. | In house using HPLC |
| Vitamin B6 (Red cell) | External lab (HPLC with fluorimetric detection) | HPLC system consisted of a Waters solvent delivery system and a Waters fluorimeter, Model 474. | In house using HPLC |
| Vitamin B12 | Competitive Immunoassay | Siemens | ADVIA Centaur |
| Iron | Colourometric using Ferrozine | Siemens | ADVIA Chemistry |
| Ferritin | Immunoassay (Sandwich) | Siemens | ADVIA Centaur |
| Vitamin C (ascorbic acid) | stabilised Metaphosphoric Acid. Measured  fluorescence using a method adapted from Vuilleumier and Keck (1989). | BMG LABTECH | FLUOstar Optima plate reader |

## Supplementary Document 4: Supplementary results Tables

**Table S1**. Change in key DIASTOLIC Outcomes within group

| **Variable** | **Routine Care** | **MRP** |
| --- | --- | --- |
| N | 30 | 24 |
| Weight loss (kg) | -1.05 (-3.16, -0.01) | -13.55 (-15.53, -11.90) |
| BMI reduction (kg/m^2^) | -0.25 (-1.00, 0.00) | -4.75 (-5.17, -4.00) |
| SBP Change (mmHg) | -7.07 (-10.60, -3.54) | -13.00 (-21.60, -4.40) |
| DBP Change (mmHg) | -1.83 (-4.65, 0.99) | -4.67 (-9.50, 0.17) |
| HbA1c Change (mmol/mol) | -0.50 (-6.00, 1.00) | -7.50 (-13.34, -5.00) |
| LV mass/volume Change (g/ml) | 0.02 (-0.01, 0.05) | -0.03 (-0.06, -0.01) |
| Aortic distensibility Change (mmHg-1x10^-3^) | 0.51 (-0.20, 1.21) | 0.90 (0.38, 1.41) |

**Table S2**. Micronutrient reference ranges

| **Micronutrient** | **Reference range** |
| --- | --- |
| Vitamin B_1_^(4)^ | 66.5-200 nmol/L |
| Vitamin B_6_ ^(4)^ | 35.2-110.1 nmol/L |
| Vitamin B_12_ ^(4)^ | 220-700 ng/L |
| Vitamin C ^(5)^ | 28–40 μM adequate/ > 40 μM optimal |
| Vitamin D ^(4)^ | >50 nmol/L |
| Iron ^(4)^ | 14-28μmol/L |
| Ferritin ^(4)^ | Male: 23-540μm/L Female: 10-420μg/L |

**Table S3**. Correlations between the change in vitamins and previously observed changes in cardiovascular structure/function following the MRP

|  | **Change in LV Mass: Volume** | **Change in mean Aortic Distensibility** |
| --- | --- | --- |
| **Micronutrient** | **Correlation Co-efficient (P value)** | |
| Vitamin B_1_ (nmol/L) | -0.10 (0.43) | 0.04 (0.73) |
| Vitamin B_6_ (nmol/L) | -0.09 (0.49) | 0.10 (0.42) |
| Vitamin D (nmol/L) | -0.2 (0.09) | 0.06 (0.63) |
| Vitamin C (µmol/L) | -0.12 (0.32) | 0.22 (0.07) |

**Table S4**. Associations between the change in vitamins and previously observed changes in cardiovascular structure/function following the MRP

| Δ LV mass: Volume Ratio |  | | | |  | | | |  | | |
| --- | --- | --- | --- | --- | --- | --- | --- | --- | --- | --- | --- |
|  | **ΔVitamin B_1_** | | | | **ΔVitamin** **B_6_** | | | | **ΔVitamin D** | | |
|  | **β** | | **95% CI** | **P** | **β** | | **95% CI** | **P** | **β** | **95% CI** | **P** |
|  | -0.37 | | -0.00, 0.00 | 0.09 | 0.05 | | -0.00, 0.00 | 0.83 | -0.14 | -0.06, 0.03 | 0.54 |
|  | **ΔVitamin C** | | | | |  |  |  |  |  |  |
|  | **β** | **95% CI** | | **P** | |  |  |  |  |  |  |
|  | 0.052 | -0.001, 0.001 | | 0.814 | |  |  |  |  |  |  |
| Δ Aortic Distensibility |  | | | |  | | | |  | | |
|  | **ΔVitamin B_1_** | | | | **ΔVitamin B_6_** | | | | **ΔVitamin D** | | |
|  | **β** | | **95% CI** | **P** | **β** | | **95% CI** | **P** | **β** | **95% CI** | **P** |
|  | 0.38 | | -0.002, .037 | 0.08 | 0.219 | | -0.01, 0.03 | 0.328 | 0.282 | -0.34, 1.48 | 0.204 |
|  | **ΔVitamin C** | | | | |  |  |  |  |  |  |
|  | **β** | **95% CI** | | **P** | |  |  |  |  |  |  |
|  | 0.28 | -0.020, 0.023 | | 0.901 | |  |  |  |  |  |  |

**Table S5**. Correlations between the change in vitamins and

changes in other cardiovascular structure/function parameters following the MRP

|  | Δ GLS | Δ E/e’ | Δ CircPEDSR |
| --- | --- | --- | --- |
| **Micronutrient** | **Correlation co-efficient (P value)** | | |
| Vitamin B_1_ (nmol/L) | -0.19 (0.39) | -0.16 (0.55) | 0.27 (0.22) |
| Vitamin B_6_ (nmol/L) | -0.26 (0.25) | -0.21 (0.43) | 0.29 (0.19) |
| Vitamin D (nmol/L) | -0.22 (0.32) | -0.256 (0.34) | -0.03 (0.90) |
| Vitamin C (µmol/L) | 0.09 (0.69) | 0.20 (0.44) | 0.07 (0.74) |

References

1. guidance NIfHaCE. <https://www.nice.org.uk>

2. Authority EFS Dietary Reference Values for the EU. <https://efsa.gitlab.io/multimedia/drvs/index.htm>

3. Carlo Agostoni RBC, Susan Fairweather-Tait, Marina Heinonen, Hannu Korhonen, Sébastien La Vieille, Rosangela Marchelli, Ambroise Martin, Androniki Naska, Monika Neuhäuser-Berthold, Grażyna Nowicka, Yolanda Sanz, Alfonso Siani, Anders Sjödin, Martin Stern, Sean (J.J.) Strain, Inge Tetens, Daniel Tomé, Dominique Turck and Hans Verhagen. (2015) Scientific Opinion on the essential composition of total diet replacements for weight control. *EFSA Journal* **13**, 52.

4. NHS UHL (2020) Blood Sciences User Handbook. In *A user guide for UHL Blood Science Pathology Services*, vol. IN5501, pp. 137 [NH Service, editor].

5. Hampl JS, Taylor CA, Johnston CS (2004) Vitamin C deficiency and depletion in the United States: the third national health and nutrition examination survey, 1988 to 1994. *American journal of public health* **94**, 870-875.
